# Development and application of a hybrid implementation research framework to understand success in reducing under-5 mortality in Rwanda

**DOI:** 10.12688/gatesopenres.13214.1

**Published:** 2021-03-29

**Authors:** Lisa R. Hirschhorn, Miriam Frisch, Jovial Thomas Ntawukuriryayo, Amelia VanderZanden, Kateri Donahoe, Kedest Mathewos, Felix Sayinzoga, Agnes Binagwaho

**Affiliations:** 1 Department of Medical Social Sciences, Northwestern University Feinberg School of Medicine, Chicago, IL, 60611, USA; 2 University of Global Health Equity, Kigali, 6955, Rwanda; 3 Maternal, Child, and Community Health Division, Rwanda Biomedical Center, Kigali, 7162, Rwanda

**Keywords:** implementation research, evidence-based interventions, under-5 mortality, Rwanda, framework, amenable mortality

## Abstract

**Background**: We describe the development and testing of a hybrid implementation research (IR) framework to understand the pathways, successes, and challenges in addressing amenable under-5 mortality (U5M) – deaths preventable through health system-delivered evidence-based interventions (EBIs) – in low- and middle-income countries (LMICs).

**Methods**: We reviewed existing IR frameworks to develop a hybrid framework designed to better understand U5M reduction in LMICs from identification of leading causes of amenable U5M, to EBI choice, identification and testing of strategies, work to achieve sustainability at scale and key contextual factors. We then conducted a mixed-methods case study of Rwanda using the framework to explore its utility in understanding the steps the country took in EBI-related decision-making and implementation between 2000-2015, key contextual factors which hindered or facilitated success, and extract actionable knowledge for other countries working to reduce U5M.

**Results**: While relevant frameworks were identified, none individually covered the scope needed to understand Rwanda’s actions and success. Building on these frameworks, we combined and adapted relevant frameworks to capture exploration, planning, implementation, contextual factors in LMICs such as Rwanda, and outcomes beyond effectiveness and coverage. Utilizing our hybrid framework in Rwanda, we studied multiple EBIs and identified a common pathway and cross-cutting strategies and contextual factors that supported the country’s success in reducing U5M through the health system EBIs. Using these findings, we identified transferable lessons for other countries working to accelerate reduction in U5M.

**Conclusions**: We found that a hybrid framework building on and adapting existing frameworks was successful in guiding data collection and interpretation of results, emerging new insights into how and why Rwanda achieved equitable introduction and implementation of health system EBIs that contributed to the decline in U5M, and generated lessons for countries working to drop U5M.

## List of Abbreviations

CFIR       Consolidated Framework for Implementation Research

CHW       Community health worker

DHS        Demographic and Health Surveys

EBI         Evidence-based intervention

EPIAS     Exploration, Preparation, Implementation, Adaptation, Sustainment

EPIS        Exploration, Preparation, Implementation, Sustainment

ERIC       Expert Recommendations for Implementing Change

IHME      Institute for Health Metrics and Evaluation

IMCI       Integrated management of childhood illness

IR           Implementation research

KI           Key informant

LMIC      Low- and middle-income country

MDG       Millennium Development Goal

M&E       Monitoring and evaluation

MOH        Ministry of Health

PCV         Pneumococcal vaccine

U5M        Under-5 mortality

## Contribution to the literature

- Implementation research frameworks are important for informing studies of successes and challenges in evidence-based intervention implementation, but typically reflect strategies and factors more relevant to high-income settings than low- and middle-income countries (LMICs).
- Building on existing frameworks, we developed a hybrid framework to inform the study of the strategies and context and outcomes of LMICs implementing EBIs to reduce amenable deaths in children under 5.
- The hybrid framework was effective when applied to understand the successes and challenges of Rwanda in dropping under-5 mortality and emerged lessons including a five-step path which other countries can adapt and adopt to accelerate their work to achieve success in saving more lives.

## Background

Over the past 75 years, the world has witnessed remarkable progress in the development of evidence-based interventions (EBIs) proven to reduce morbidity and mortality from the leading causes of under-5 mortality (U5M) globally. These include biomedical interventions, such as vaccinations and oral rehydration salts, as well as strategies for delivery of these interventions through the integrated management of childhood illness in facilities and through community health workers. The United Nations Millennium Development Goal (MDG) 3 called for reduction of U5M by two-thirds between 1990 and 2015. In response, low- and middle-income countries (LMICs) prioritized U5M reduction through the introduction and strengthening of relevant EBI implementation as well as by addressing other direct and contributing causes of mortality, such as female disempowerment, stunting, and poor access to reproductive healthcare services ^1^. While U5M rates dropped by 53% from 1990 to 2015 globally, only 70 (36%) of the 193 UN Member States achieved the U5M goal, with 98.6% of U5 deaths occurring in LMICs in 2015 ^2, 3^.

Many of the EBIs known to be effective in reducing U5M were either not fully implemented or implemented with variable quality (effective coverage) in many LMICs. As a result, a significant proportion of deaths among children under 5 in these countries were preventable through a stronger health system that was able to provide quality care equitably (defined as amenable death) ^4^. Insights into successes and challenges in how EBIs were chosen, adapted, implemented, and sustained are often missing from available reports and publications, which often focus on coverage and effectiveness. Implementation research (IR) is defined as “the scientific study of the use of strategies to adopt and integrate evidence-based health interventions into clinical and community settings to improve individual outcomes and benefit population health” ^5^. The discipline was developed in response to this gap between awareness of EBIs and their effective and equitable implementation. IR can accelerate more effective planning, implementation, scaling, and sustainment of EBIs and policies, taking into account the contextual factors that influence outcomes ranging from coverage, to acceptability, to effectiveness. IR provides a set of methods for policymakers, implementers, and researchers to design and learn how to better choose and implement EBIs through strategies devised to move evidence from research into practice ^6, 7^.

Interest and use of IR in LMICs has grown in recent years, supported by publications illustrating the value of applying these methods to understand implementation successes and challenges in high-, middle-, and low-income countries ^8– 11^. However, a number of publications have identified where adaptations are needed for these frameworks which were largely developed in high income settings ^12– 14^. For example, a recent review of the use of IR in LMICs found a need for its expanded use in order to understand scale-up, sustainability, and implementation in more real-world settings ^8^. In addition, existing IR frameworks with adaptations are needed to understand the full path from decisions at the policy level through to sustainable scaling and the contextual factors specific to public sector health systems in LMICs and ensure the focus on equity, central to achieving Universal Health Coverage (UHC) ^13, 14^.

We describe the development and testing of an IR framework building on existing frameworks to guide the study of pathways, successes, and challenges in addressing amenable U5M – those deaths preventable through health system-delivered EBIs – in LMICs. The application of the framework was tested through a mixed-methods case study of Rwanda, a country well known for its remarkable success in substantially reducing U5M from 2000 to 2015 and improving or achieving equity in many of these areas Table 1 ^15^. The hybrid framework was used to inform the mixed methods case study design and extraction and analysis of implementation strategies and outcomes and key contextual factors to understand the process, successes, and challenges in Rwanda from identification of the causes of death, to EBI choice, through to work on sustainability and scaling. The case study was conducted as part of the Exemplars in U5M project and is available in full at https://www.exemplars.health/. In this paper, we describe the framework and its value in identifying key lessons in how and why Rwanda achieved this success to create potentially transferable and actionable knowledge to inform other countries working to reduce amenable U5M.

**Table 1.** Changes in under-5 mortality and evidence-based intervention coverage and equity gaps in Rwanda between 2000 and 2014. *(Source: Rwanda DHS 2000, 2005, 2010, and 2014–15 and DHS STATcompiler)*.

Mortality		2000	2005	2010	2014	Wealth equity gap in 2000	Wealth equity gap in 2014	Absolute change in wealth equity gap 2000–2014
U5M/1000 live births		207	152	76	50	73	45	-28
NMR/1000 live births		50	37	27	20	19	11	-8
**U5 Cause of** **Death**	**Evidence-Based** **Intervention**							
Lower Respiratory Infections	Care-seeking for pneumonia	15.5%	27.9%	50.2%	53.9%	20	20	0
	Vaccination: 3 doses of PCV	NA	NA	NA	94.7%	N/A	5%	N/A
	Vaccination: Hib	NA	NA	92.9%	98.2%	N/A	3%	N/A
Diarrheal Diseases	Oral rehydration therapy	20.2%	29%	36.6%	34.7%	11%	12%	1%
	Vaccination: 3 doses of rotavirus				94.7%	N/A	5%	N/A
	Care-seeking for diarrhea	15%	14.4%	41%	45%	9%	18%	9%
Malaria	Insecticide-treated nets		12.6%	69.6%	67.7%	N/A	30%	
	Care-seeking for fever	8.7%	31.3%	44.8%	50.1%	10%	24%	14%
	Treatment of children with fever by ACT *			4.0%	11.2%	N/A	-5%	N/A
	Prompt treatment of children with fever by ACT			2.6%	7.4%	NA	-1%	N/A
Measles	Vaccination: Measles	86.9%	85.6%	95.0%	95.2%	4%	6%	2
HIV	HIV counseling during antenatal care		55.8%	90.6%	93.0%	N/A	3%	
	HIV testing during antenatal care		21.5%	94.5%	97.9%		3%	
Other vaccine preventable diseases	Full vaccination coverage with 3 doses DPT, 3 doses polio, measles and BCG	76.2%	75.5%	90.3%	92.7%	6%	8%	5%
Neonatal-specific intervention’s	Antenatal care: 1+ visits by a skilled provider	92.5%	94.4%	98.0%	99.1%	N/A	N/A	N/A
	Antenatal care: 4+ visits by a skilled provider	10.4%	13.3%	35.4%	43.9%	N/A	N/A	N/A
	Antenatal care: 1st antenatal visit in the 1st trimester	4.7%	7.9%	38.2%	56.1%	N/A	N/A	N/A
	Vaccination: 1+ doses of tetanus toxoid during pregnancy	64.8%	63.4%	76.5%	79.6%	N/A	4%	N/A
	Delivery in a health facility	26.5%	28.2%	68.9%	90.7%	43%	13%	-30
	Delivery attended by skilled provider	26.7%	28.4%	69.0%	90.7%	N/A	13%	N/A
	Delivery by Cesarean- section	2.1%	2.9%	7.1%	13.0%	N/A	13%	N/A
	Postnatal care: Postnatal visit for baby within 2 days of birth	2.9%	3.7%	4.7%	19.3%	N/A	-1%	N/A
	Postnatal care: Postnatal visit for mother within 2 days of birth			17.6%	43.0%	N/A	10%	N/A

Wealth equity gap calculated as difference between the wealthiest quintile and lowest wealth quintile. N/A: not available as equity gap not able to be calculated due to no wealth disaggregation the DHS reports for some indicators. * ACT: artemisinin-based combination therapy.

## Methods

### Development of the amenable U5M implementation research framework

We reviewed existing implementation research frameworks focusing on those applied in LMICs to study implementation strategies, identification of contextual factors, and implementation outcomes ^8, 16– 19^. Relevant frameworks were identified to develop a hybrid framework, which was adapted during the case study process to reflect emerging areas where changes were needed.

### EBIs to reduce amenable U5M

Reviewing existing guidelines and literature from the MDG efforts, we identified EBIs known to reduce the most common causes of U5M in LMICs for infants and children, and for neonates during the three periods of risk (antenatal, peripartum, and postpartum), to guide the exploration of implementation strategies, contextual factors, and implementation outcomes (Extended data Tables 1a and 1b) ^20– 22^. These EBIs also guided evidence review and key informant selection.

### Extraction and analysis of implementation pathways, strategies, outcomes, and contextual factors

We used a mixed methods case study to leverage existing quantitative data on selected implementation outcomes (reach, fidelity, adoption) and equity with the qualitative sources describing strategies, contextual factors and selected outcomes (acceptability, feasibility). The Standards for Reporting Implementation Studies (StaRI) were used where relevant and is included in the Extended data ^22, 23^.

***Desk review***. The team reviewed available information and published data on the rates and progress of U5M broadly and amenable causes of U5M, and the uptake and implementation of specific EBIs, including policies and strategies. Initial review research was performed through MEDLINE (PubMed) and Google Scholar using the following search terms: “child mortality” or “under-5 mortality” and Rwanda. Further searches included terms for specific EBIs (ex. “pneumococcal vaccination,” “oral rehydration salts,”), causes of death (ex. “malaria,” “measles,” “HIV”), or contextual factors (e.g. “community health workers,” “female empowerment”). Other sources included publicly available datasets and reports (Demographic and Health Survey, Countdown 2015, and existing reviews of U5M reduction work in Rwanda) ^20, 24– 27^. The review was limited to amenable causes of death from the neonatal period to early childhood, including EBIs during pregnancy and childbirth deemed critical for child survival ^20, 24– 27^. The review was limited to amenable causes of death from the neonatal period to early childhood, including EBIs during pregnancy and childbirth deemed critical for child survival. Important interventions that contributed to U5M reduction beyond the specific health system EBIs including health system strengthening, education, female empowerment, reproductive health, poverty reduction, improving water and sanitation, and programs designed to improve nutritional status beyond management of acute severe malnutrition were captured as potentially important contextual factors. However, in-depth reviews of the implementation of these interventions not directly led by the health sector were beyond the scope of the research. The desk review was an iterative process, with additional searches conducted as needed and informed by the primary research described below.

***Key informant interviews***. In collaboration with our in-country consultant (FS), we identified 16 key informants (KIs) who had been involved throughout the process of identifying the burden of the target cause of death and selecting, implementing, and working to sustain the targeted EBIs and U5M reduction more broadly before and during the period of focus (2000–2015). These KIs included current and former Ministry of Health employees responsible for overall U5M efforts, or specific diseases or intervention areas. We also interviewed KIs from implementing non-governmental organizations, multilateral organizations, and donor organizations who had been involved in partner-supported or partner-led activities. Some KIs represented more than one viewpoint based on their experience over the 15 years and were interviewed for each of their multiple viewpoints.

The interviews were conducted using a semi-structured guide (see Extended data ^22^), based on the IR framework, designed to understand EBI selection; the implementation process, from exploration to preparation, implementation, and sustainment; and contextual factors contributing to successes and challenges in each stage. The interviews also helped identify additional data sources important in understanding implementation and related outcomes. Follow-up interviews were conducted as gaps or additional needs were identified. All interviews except one were led by one of the project principle investigators with one to two note-takers. The total number was not designed for saturation but instead for covering the EBIs and was also limited by time and resources. Informed consent was obtained from all interview participants, and all recordings and transcripts were de-identified and stored in password-protected folders and destroyed once the interview coding was completed. The results were disseminated to the Ministry of Health and partners.

Following the close of the interview, notes were combined and recordings (if allowed) were used to clarify areas as needed. Thematic coding of interviews using inductive and deductive approaches was done by one of the researchers and reviewed by one of the principal investigators for accuracy, with discussion for differences. *A priori* codes were organized from the framework for EBIs, implementation outcomes, and context, with implementation strategies informed by the Expert Recommendations for Implementing Change (ERIC) framework ^28^ and additional codes added as emerging themes were identified.

Additional analyses from the International Center for Equity in Health and geospatial mapping from the Institute for Health Metrics and Evaluation (IHME) were used to understand changes in equity indices for mortality and EBI coverage.

### Ethical considerations

The study was determined to be non-human subjects research by the Rwandan National Ethics Committee and Northwestern University reflecting the scope and focus.

## Results

### Framework

**Figure 1.**
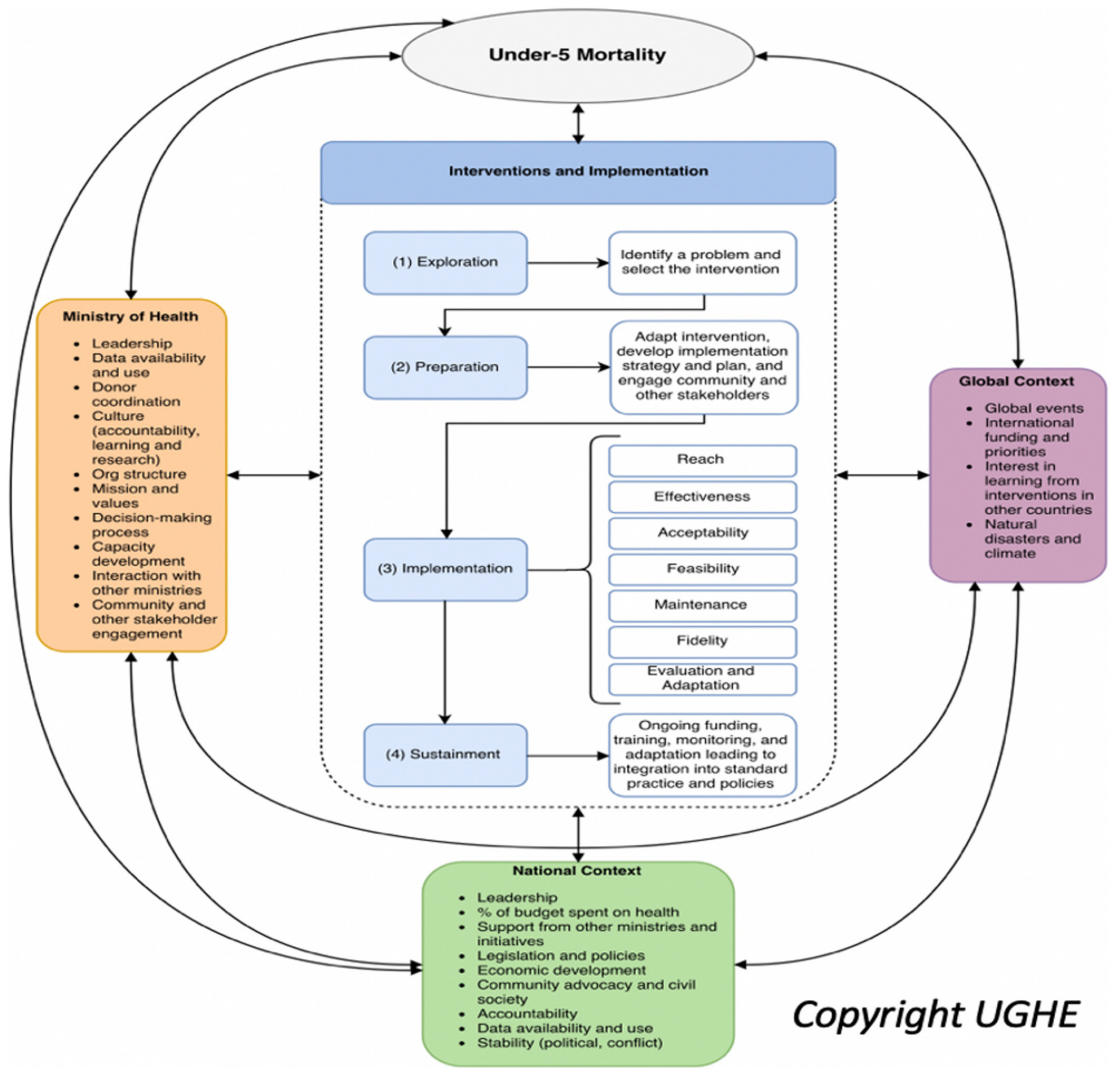
Hybrid Framework for Understanding Interventions to Reduce Under-5 Mortality. Reproduced with the permission of UGHE.

We found that no single framework covered the scope of the work from identification of EBIs to target leading causes of U5M, to selection and testing of strategies to achieve sustainability at scale, understanding the full range of potential contextual factors and exploring outcomes beyond the traditional coverage and effectiveness measures used in evaluation of health systems. We therefore combined and adapted a number of existing frameworks to synthesize a framework designed to guide our work to understand implementation of and outcomes of EBIs to reduce amenable U5M ( Figure 1). The frameworks identified as appropriate for guiding our work included:

1.     
*Exploration, Preparation, Implementation, Sustainment (EPIS)* ^16^: EPIS covers four key steps of the implementation process from initial decision-making through work to achieve long-term change. Within each phase, Aarons identifies potential contextual factors that may influence success.
2.     
*RE-AIM* ^29^: The framework identifies outcomes in Reach (coverage), Effectiveness, Adoption of the EBI, Implementation (fidelity, time, cost, and adaptations made), and Maintenance (institutionalization into routine care and policies and long-term impact).
3.     
*Consolidated Framework for Implementation Research (CFIR)* ^17^: CFIR was designed to understand contextual factors that influence the success or failure of implementation of an EBI.
4.     We also expanded the outcomes of RE-AIM by incorporating relevant implementation outcomes key to achieving overall effective coverage and equity ^18^.

The framework was revised as needed as new themes emerged during the Rwanda case study. For example, we recognized that adaptation was an important step during implementation and was sufficiently crucial that we included it as an explicit step rather than as an embedded implementation strategy. This led to the transformation of the four-step EPIS implementation process into the five-step EPIAS implementation process. In addition, this adapted framework expands the levels at which we captured contextual factors, looking at global, national and subnational, ministry of health and health system, and community/family/individual levels. As the arrows indicate in Figure 1, our framework also explicitly calls out the interplay and non-siloed nature of contextual factors and emphasizes the need to consider the multifaceted nature of these factors important in analyzing health sector implementation outcomes as well as broader influences at the global, national and health system levels. These factors included those which could be leveraged to support implementation and those that represented barriers, requiring direct or indirect action to address, adaptation of implementation strategies at the local or national levels, or representing reasons for challenges in achieving targeted outcomes.

### Development of a theory of change

**Figure 2.**
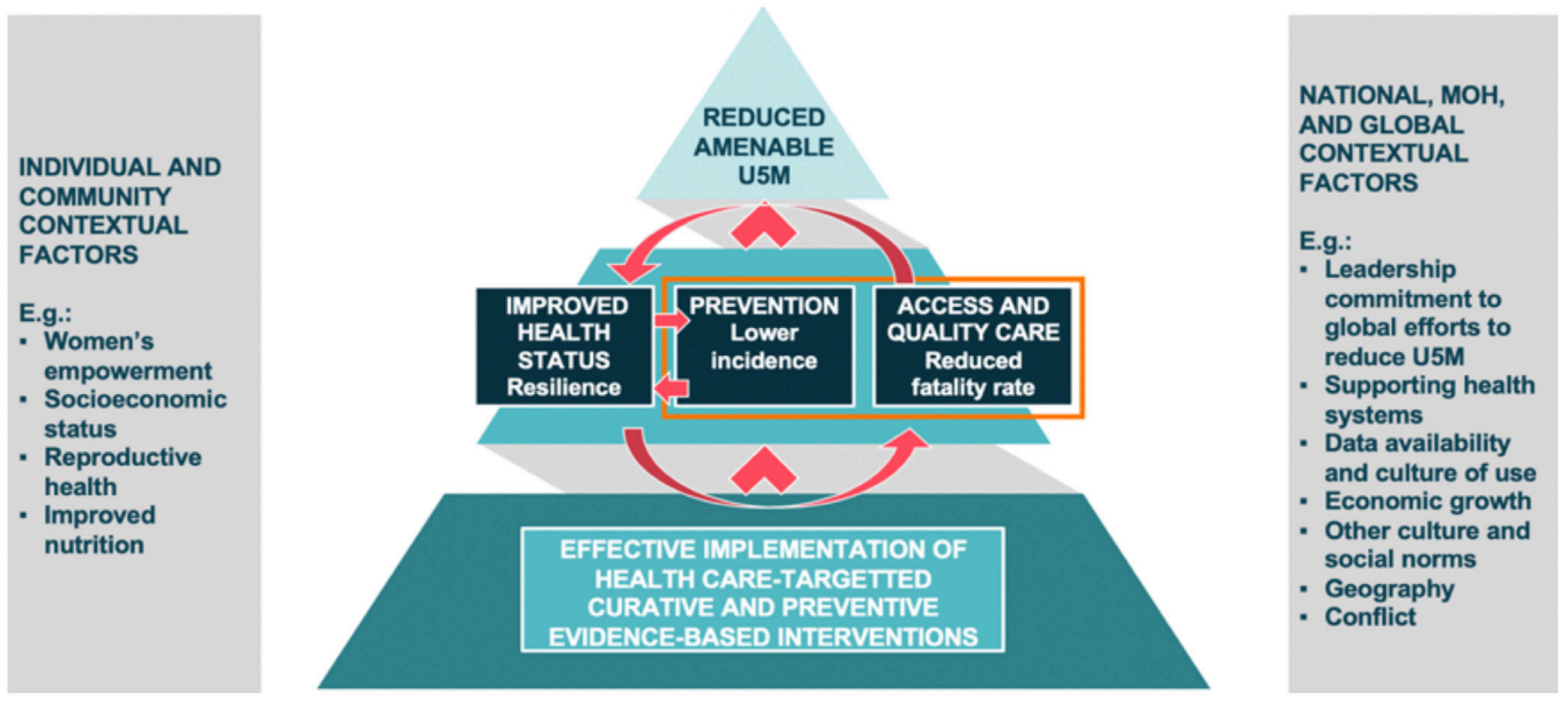
Theory of Change for Reduction of Amenable Under-5 Mortality.

To put the work to reduce amenable U5M within the broader work to reduce U5M, we also created a theory of change model ( Figure 2) including the health systems EBIs which were the focus of our research (outlined) and other interventions which could directly or indirectly improve the health status of the children (resiliency), reduce the risk of falling ill or dying during an illness, or improve the ability to access care. These included interventions to improve nutrition, water and sanitation, female empowerment, and economic status. The theory of change takes into account the contextual factors at the four levels that can contribute to the reduction of amenable U5M and U5M more broadly. These factors can influence the implementation of EBIs, health resilience, prevention of disease, and access and quality care, all of which can contribute to or hamper work to reduce amenable U5M.

### Applying the framework to understand U5M reduction in Rwanda

We used the framework to develop the key informant guides and overall analysis plan. The framework was then used to organize the information from the desk review and do an inductive and deductive thematic analysis of the KI interviews. The goals were to extract understanding of how and why Rwanda was able to successfully implement health systems EBIs known to reduce amenable U5M, and where challenges remained in implementation, sustainability, and overall U5M reduction. No software was used for this first qualitative coding.

### Implementation steps and strategies

Implementation of all the EBIs followed the steps from the hybrid framework (EPIAS), starting with **Exploration** to determine the relative burden of an underlying cause of U5M and identify potential interventions. The **Preparation** step was carefully conducted and included a range of strategies including: stakeholder engagement; identifying contextual factors that either needed to be addressed (ex. supply chain strengthening or human resources) or that influenced the choice of implementation strategy (ex. task sharing); identifying and collecting any new evidence needed to inform strategies and adaptation; determining funding needs and sources; strengthening needed systems and developing or adapting national policies and protocols to drive systematic adoption; and integration of data needs for monitoring and evaluation (M&E) into routine information systems when possible and ensuring accountability for EBI delivery. **Implementation** then followed, with the pace of implementation based on EBI rollout needs and readiness. For example, vaccines were rapidly implemented at national scale while others, such as integrated management of childhood illness (IMCI), were rolled out as ongoing learning occurred.

The implementation of all of the EBIs included **Adaptation** starting soon after initial Implementation step, reflecting evidence emerging from ongoing monitoring and evaluation or other data sources reflecting how the strategies were put into practice, new global evidence (ex. rapid testing for malaria), or identification of areas where gains in coverage were either not reaching the most vulnerable or diseases, such as malaria, were reemerging. In Rwanda, the work for **Sustainment** was often included early in the steps through strategies including integration into national standards, ensuring access to long-term funding, fostering a culture supportive of M&E, and inclusion into training and supervision. Throughout these five steps, a number of implementation strategies were identified across the different EBIs ( Table 2).

**Table 2.** Common Implementation Strategies by Exploration, Preparation, Implementation, Adaptation, and Sustainment Stages in Rwanda.

 Strategy effectively implemented  Strategy implemented with variable success  Strategy not implemented				
Implementation strategy	Exploration	Preparation	Implementation and Adaptation	Sustainment
Donor and implementing partner coordination				
Focus on equity				
Engagement of key national stakeholders and partners				
Engagement of key international stakeholders and partners				
National leadership and accountability				
Building and strengthening primary healthcare systems				
National prioritization of health				
Rapid and early adoption of innovations				
Development of national policies, guidelines, and standards (including adaptation of global protocols/ guidelines)				
Community engagement and education				
Data generation by in-country institutions and use				
Data use for understanding gaps, prioritization, implementing, adapting				
Multi-sector approach				
Rapid scale-up				
Small-scale testing				
Supportive supervision and mentoring for quality				
Building on community health worker program and community-based care delivery				
Human resources for health expansion				
Leveraging and strengthening existing systems (using to implement a strategy)				
Task-shifting				
Government financing				

***Implementation outcomes***. We found that Rwanda implemented almost all of the EBIs identified as effective in reducing U5M (Extended data Table 1a ^22^) in LMICs with varying coverage but high levels of equity ( Table 1, Extended data Figure 1 ^22^). Neonatal mortality reduction was slower and work to implement a number of the neonatal-specific EBIs (Extended data Table 1b ^22^) became a greater focus towards the end of the study period ( Table 1).

However, extraction of many implementation outcomes was limited by the available evidence from the primary and secondary resources. The most common outcomes we found from the desk review and KI interviews included feasibility, penetration/reach, and adoption. Sustainability outcomes were found for a portion of the EBIs and defined as maintaining high coverage rates from initial implementation through the end of the study period and strategies associated with sustainability including incorporation into national policies and budgets. Fidelity and cost were infrequently found except at subnational levels from targeted studies.

***Cross-cutting contextual factors***. We identified cross-cutting contextual factors that were critical to successful implementation of many of the EBIs in Rwanda (Extended data Table 2 ^22^. Equally important, a number of these also contributed to the decline in U5M by addressing causes not directly related to health. System interventions were important facilitating contextual factors throughout the five steps of EPIAS and represented the four areas from the framework (global, national, health system, and community). These included national leadership and commitment (in policy and finances) to health; a culture of data use for learning and decision-making tied to accountability at all levels; and, importantly, the existence of standards for coordination, collaboration, and communication within the health sector, across ministries, with local leaders, and between government bodies, donors, and implementing partners. Other facilitators included the availability of donor support and in-country implementing partners. The underlying health system design, with its commitment to primary care and decentralization, along with the development of a robust community health worker (CHW) program were also identified as facilitators, building on an underlying focus on equity. Resiliency of the family and child were also strengthened through national economic development, female empowerment through education and policy, development of a national health insurance fee to the poorest sectors and emerging work to address nutrition and stunting.

There were also a number of contextual factors which represented challenges in Rwanda, including gaps in components of the health system – such as ambulances and advanced equipment for the care of sick and low birthweight neonates – and cadres of human resources for health.

### Example of applying the framework to understand EBI implementation: the case of pneumococcal vaccine introduction

We use the introduction of pneumococcal vaccine (PCV) to illustrate the results of employing the framework to understand the strategies, implementation outcomes, and contextual factors that explain the steps taken and overall results. We highlight examples of strategies and facilitating factors which emerged during the five steps outlined in our framework.

### Exploration

Rwanda decided to rapidly move forward with PCV, based on the announcement by GAVI of the availability of grants for PCV; recommendations from WHO; and identification of pneumonia as the leading cause of death among children under 5 in Rwanda ( *strategies*: use of external evidence and local data use for decision-making; *factors*: data availability, culture of data use) ^30– 34^. This decision was led by the Rwandan Ministry of Health (MOH) and supported by the Vaccine Steering Committee, made up of MOH officials, one of whom chaired it, and national and international partners ( *strategies*: national leadership, multisectoral and donor coordination; *factors*: national commitment to heath, culture of coordination and intersectoral collaboration) ^35^.

### Preparation

Supported by both the MOH and international partners (ex. GAVI, UNICEF, and USAID), Rwanda first performed a nationwide evaluation of the existing cold chain, which was used to address infrastructure gaps ( *strategies*: integration into existing systems, leveraging global resources; *factors*: focus on primary care systems, donor fund availability). Rwanda also developed national training modules, implemented a cascading train-the-trainers model, revised vaccination guidelines and reporting forms, developed key messages for communities, and conducted a needs estimate ( *strategies*: training with national protocols, integrating into existing monitoring systems, community engagement and education; *factors*: community trust in the healthcare system, existing monitoring and evaluation and health information systems) ^33^. The MOH procured the very high temperature incinerators (>1200°C) needed for destruction of prefilled glass-containing syringes ^36^. The MOH secured in the national budget, the counterpart fund for GAVI ( *strategy*: engagement of key national stakeholders and partners) and chose to immediately roll out implementation nationwide due to confidence in their pre-implementation evaluation of the project, safety, and equity profiles of the vaccine, and a commitment to equity.

### Implementation

Implementation of PCV started in 2009, with Rwanda becoming the first developing country to introduce the pneumococcal vaccine ^37^. It rolled out province-by-province as planned and was completed within five months of the start date. The implementation strategy ensured equity by using motorcycles and cold boxes to deliver vaccines and leveraging CHW data to determine which areas needed additional vaccination days. A number of implementation outcomes were achieved, facilitated in part by the strategies chosen ( Table 3).

**Table 3.** PCV Implementation Strategies and Selected Implementation Outcomes.

	Implementation Strategy	Implementation Outcomes
**Acceptability**	Sensitization of local leaders, teachers, and traditional healers; outreach by CHWs on risk and benefits of vaccinations and through newspapers, radio, and television based on messages created by MOH ^33, 35^.	High acceptability because Rwanda’s “population trusts very much their government and what they are doing and that [the MOH’s] main interest is our beneficiaries.” (KI)
**Feasibility**	The supply chain and M&E were successfully adapted with data use to identify gaps in coverage.	No stockouts occurred during the vaccine rollout and vaccinations were able to be implemented ^33^.
**Fidelity**	MOH officials performed regular supervisory visits at every health center and provided feedback ^33^. UNICEF, the Centers for Disease Control and Prevention (CDC), the American Red Cross, and USAID/MCHIP joined local evaluators to organize the post introduction evaluation.	The rollout was completed as planned within five months.
**Effectiveness and Reach**	Infrastructure assessment and investment; cascade training for vaccinators; needs estimation by CHWs; nationwide, province-by-province, rolled out over five months.	PCV coverage quickly increased to 97% by 2010. There was a 53% reduction in child hospitalization due to pneumonia after introduction of PCV ^38, 39^.

### Adaptation

In 2011, in response to high cold storage and incineration temperature requirement for PCV-7, Rwanda switched from using the PCV-7 to the PCV-13 vaccine which also provided protection against six more bacterial strains. These changes allowed for adoption of new vaccines, which had similar storage and disposal needs, with little increase in infrastructure and informed the strategy for introduction of other new vaccines in Rwanda in the future ^33, 35^.

### Sustainment

Full vaccination rates with PCV remained consistently high from introduction through the end of the study period and beyond, with coverage at 97–98% since 2010. Strategies that were identified as supporting this sustainment included integration by the MOH into its standard pediatric vaccine schedule and regular data use through monthly monitoring data, which are analyzed at the central level with feedback to address coverage gaps ^35^, and continuation of the national funding for PCV, which has been included in the annual national budget since its introduction in 2009.

Rwanda has been highlighted as a country that leads in work to drop U5M and we were able to surface a number of lessons that are transferable to other countries that wish to accelerate their work to reduce U5M. These emerged from the successful strategies directly related to implementation, as well as from contextual factors, which can be modified to facilitate successful implementation outcomes ( Table 4).

**Table 4.** Transferable Lessons for Countries Looking to Accelerate Decline in U5M Through More Effective EBI Implementation.

• Ensure accountability at all levels and engage community to support
• Build capacity of implementers and policymakers in the ministry and locally
• Change the culture of data use to include training, increased data use and quality, and linkages to accountability systems
• Coordinate donor and implementing partner funds and activities to follow the national vision and strategy, leveraging these important resources
• Provide support to strengthen leadership at all levels through delegation of responsibility accompanied by the accountability
• Create laws, policies, and regulations needed for effective quality implementation, and enforce them to ensure quality and delivery
• Engage the community and civil society at all levels and in meaningful ways, including through bylaws and national regulation
• Invest in health systems and inputs, including physical accessibility and quality broadly and leverage them for specific EBIs
• Ensure financial accessibility and protection through systems designed to ensure equity
• Engage the private sector and nongovernmental and faith-based organizations as key partners in care delivery
• Plan for equity from the beginning

## Discussion

We found that by building on and combining established IR frameworks and adapting based on themes that emerged during our case study, we were able to develop a hybrid framework that was successfully used to guide how we prioritized research areas and analysis to extract knowledge of how Rwanda chose, implemented, and sustained EBIs to contribute to their remarkable drop in U5M, contributing or hindering actors and the implementation outcomes achieved including a focus on equity as an explicit outcome. Adaptations included adding in an additional step of adaptation after implementation because of its prominence as an explicit stage in the pathway to successful implementation, and expansion of the levels at which we captured contextual factors. This successful use of IR builds on existing work in other countries in Africa, which have also found the value in understanding how and why interventions were successfully implemented and if outcomes including and beyond effectiveness were achieved ^10, 11, 40^. Our finding of a need for adaptations of existing IR frameworks reflects findings from other researchers working to increase IR use and effectiveness in LMIC. For example, the recent review of uses of the CFIR framework found modifications needed to be more applicable for use in these settings with similar results from work using RE-AIM ^12, 13^.

Applying this framework also supported the extraction of implementation strategies that were common across many if not all of the EBIs. These often went beyond the categories outlined in guidelines for categorizing strategies, such as ERIC ^41^, potentially reflecting the use of IR in a different context than the ones for which ERIC was designed. Identifying these strategies was important as they represent opportunities for learning and adoption in other countries, although differences in contextual factors would need to be addressed. For example, one cross-cutting strategy was coordination of donors and partners but led by a national plan and vision and stakeholder engagement throughout the five steps of EPIAS. These strategies were identified as critical for the feasibility, fidelity, and reach of the EBIs, as well as an ability to continue focusing on equity. These strategies built on contextual factors in a number of the four domains including strong national and ministry leadership and a culture of accountability, factors which may need to be strengthened in other settings. Another key strategy was integrating new EBIs into existing systems, including primary healthcare. The success of these strategies was facilitated by the national commitment to primary healthcare, including expanding health facilities and a robust community health worker program. This strategy, combined with rapid data use for decision-making and planning, was also used to rapidly roll out new vaccines during the study period and beyond ^35, 42^.

The focus on contextual factors and the theory of change also allowed for exploration of interventions and other factors beyond the health system-delivered EBIs that contributed to their effectiveness and overall decline in U5M. These included female empowerment, access to family planning to improve birth spacing, economic improvement, reduction in stunting (albeit less impressive than the reduction in U5M), and other efforts. The contribution of these factors is consistent with modeling work done by IHME in Rwanda, as well as results from the Countdown 2015 studies in other countries ^43, 44^.

There are a number of limitations to our work. We relied on available quantitative data and published reports for the measurement of many of the outcomes. As noted, reports and data on key factors such as quality, cost, and reasons for variability in implementation outcomes were often missing. The key informant interviews relied on recall, so bias cannot be excluded. We also ensured confidentiality, but as in all interviews, there may have been social desirability bias, a factor we tried to limit by having the interviews conducted by investigators other than the coauthors involved in the work done in Rwanda during the study period (AB and FS). As is the case with many retrospective IR studies, there were no validated measures for outcomes such as adoption, acceptability, and reported appropriateness. Finally, we explicitly designed the study to focus on the implementation of known EBIs in a real world setting and so the attribution of the degree of contribution to the overall reduction in U5M could not be directly measured, although modeling results are available at www.exemplars.health.

## Conclusion

In conclusion, we found that using the developed U5M IR framework facilitated the identification of the implementation pathway, priority implementation outcomes, and contextual factors important in healthcare in LMICs. The case study was able to increase insights into how and why Rwanda achieved the equitable introduction and expansion of EBIs contributing to the decline in U5M. The framework has now been successfully used in five other countries (Peru, Senegal, Ethiopia, Nepal, and Bangladesh) with results available at http://www.exemplars.health. The goal of the case study and project is to create generalizable and actionable knowledge needed by other countries working to accelerate and ensure equity in their reduction of U5M. This work is also part of an ongoing movement to expand the use of IR as a powerful tool for understanding and evaluating performance, identifying contextual factors that require strategy adaptation or direct action, and revealing global promising practices, which countries can use to achieve the goal of eliminating deaths in children everywhere.

## Data availability

### Source data

The Demographic and Health Surveys data used in the current study are available from the DHS website.

Rwanda DHS 2015: https://dhsprogram.com/pubs/pdf/FR316/FR316.pdf

Rwanda DHS 2000: https://dhsprogram.com/pubs/pdf/FR125/FR125.pdf

The Institute for Health Metrics and Evaluation (IHME) Low- and Middle-Income Country Neonatal, Infant, and Under-5 Mortality Geospatial Estimates 2000–2017 Local Burden of Disease data used in the current study are available for download from IHME: http://ghdx.healthdata.org/record/ihme-data/lmic-under5-mortality-rate-geospatial-estimates-2000-2017.

### Underlying data

Data access is restricted to users with appropriate ethics approval from the committees listed in the Ethical Considerations section. A reader or reviewer may apply to the authors for access by providing a written description of background, reasons, and intended use. If the methodology does not violate the condition of informed consent under which the interview was conducted, and the proposal approved by UGHE and other relevant ethics boards, the user can obtain the data from the corresponding author, and include one of the authors in the project and analysis.

### Extended data

DRYAD: Development and application of a hybrid implementation research framework to understand success in reducing under-5 mortality in Rwanda. https://doi.org/10.5061/dryad.kh189324x ^22^

This project contains the following extended data:

-     Interview guide, accessible here: https://ughe.org/wp-content/uploads/2021/02/1.-Interview-Guide_Exemplars-U5M.pdf.
-     Tables and figures, accessible here: https://ughe.org/wp-content/uploads/2021/02/2.-Extended-Data-Tables-1a-and-1b-Table-2-Figure-1.pdf.

### Reporting guidelines

DRYAD: StaRI checklist for ‘Development and application of a hybrid implementation research framework to understand success in reducing under-5 mortality in Rwanda’ https://doi.org/10.5061/dryad.kh189324x ^22^. Accessible here: https://ughe.org/wp-content/uploads/2021/02/3.-StaRI-checklist-IS-Exemplar-framework-methods.pdf.

This work is licensed under a CC0 1.0 Universal (CC0 1.0) Public Domain Dedication license.

**Ethics approval and consent to participate:** The study was determined to be non-human subjects research by the Rwandan National Ethics Committee and Northwestern University reflecting the scope and focus.

Interviewees were informed about the goals and structure of the project, and consent for participation and for recording was obtained separately from the interview.
