# Supplementary material for: Development and application of a hybrid implementation research framework to understand success in reducing under-5 mortality in Rwanda
Source: Gates Open Res. 2021 Dec 17;5:72. Originally published 2021 Mar 29. [Version 3] doi: 10.12688/gatesopenres.13214.3 (PMC8688814; doi:10.12688/gatesopenres.13214.3)
Supplement: Peer review history

# Peer review: Reviewer response for version 3

**DOI:** 10.21956/gatesopenres.14748.r31579

**Published:** 2021-12-20

**Authors:** Ejemai Amaize Eboreime

**Affiliations:** 1 Department of Psychiatry, Faculty of Medicine and Dentistry, University of Alberta, Edmonton, AB, Canada

No further comments

Is the work clearly and accurately presented and does it cite the current literature?

Partly

If applicable, is the statistical analysis and its interpretation appropriate?

Not applicable

Are all the source data underlying the results available to ensure full reproducibility?

Partly

Is the study design appropriate and is the work technically sound?

Partly

Are the conclusions drawn adequately supported by the results?

Partly

Are sufficient details of methods and analysis provided to allow replication by others?

No

Reviewer Expertise:

NA

I confirm that I have read this submission and believe that I have an appropriate level of expertise to confirm that it is of an acceptable scientific standard.

# Peer review: Reviewer response for version 2

**DOI:** 10.21956/gatesopenres.14720.r31449

**Published:** 2021-12-08

**Authors:** Ejemai Amaize Eboreime

**Affiliations:** 1 Department of Psychiatry, Faculty of Medicine and Dentistry, University of Alberta, Edmonton, AB, Canada

I appreciate the authors' revisions and clarifications following my earlier comments. 

 I still do have some reservations. 

 Whereas the authors provide explanations/clarifications to some of my earlier concerns raised, some of these were changes not effected in the manuscript. It should be noted that the response to reviewer comments only addresses the reviewers, but without translating this clarification to the main manuscript, there is no clarification for the readers. For example, the manuscript still does not detail how and when the authors searched the literature for IS frameworks. The authors provide some explanations to the reviewer's comments, but the manuscript still does not detail this process.

Is the work clearly and accurately presented and does it cite the current literature?

Partly

If applicable, is the statistical analysis and its interpretation appropriate?

Not applicable

Are all the source data underlying the results available to ensure full reproducibility?

Partly

Is the study design appropriate and is the work technically sound?

Partly

Are the conclusions drawn adequately supported by the results?

Partly

Are sufficient details of methods and analysis provided to allow replication by others?

No

Reviewer Expertise:

NA

I confirm that I have read this submission and believe that I have an appropriate level of expertise to confirm that it is of an acceptable scientific standard, however I have significant reservations, as outlined above.

# Response

**Published:** 2021-12-10

**Authors:** Amelia VanderZanden

**Affiliations:** University of Global Health Equity, USA

**Comment**: *I appreciate the authors' revisions and clarifications following my earlier comments.*

*I still do have some reservations.*

*Whereas the authors provide explanations/clarifications to some of my earlier concerns raised, some of these were changes not effected in the manuscript. It should be noted that the response to reviewer comments only addresses the reviewers, but without translating this clarification to the main manuscript, there is no clarification for the readers. For example, the manuscript still does not detail how and when the authors searched the literature for IS frameworks. The authors provide some explanations to the reviewer's comments, but the manuscript still does not detail this process.*

**Response**: Thank you for your careful consideration. While the open peer review and response process means that all readers are able to see both the reviewer comments and the authors' responses in addition to the article, we agree that in our previous revision we did not include changes to the article beyond our response to this reviewer's comment about the rapid review for framework choice. For all other previous comments from this reviewer we have reviewed and added as needed to other previously documented responses. 

 The work on framework development was done in 2017. The authors focused on identifying implementation research frameworks which had been used in LMICs at that time and were relevant to health systems interventions. We conducted a search for “implementation science” and "LMICs" in PubMed as a rapid review. We also reviewed publications in Implementation Science in the last three years, and through outreach and discussion with experts in the field at the time. No single framework  captured the contextual factors and stages of implementation as well as implementation outcomes, so a hybrid framework which could be used across the scope of the work was developed.

 We have now expanded the Methods section text under the header, "Development of the amenable U5M implementation research framework," to include the following italicized text: " *At the start of the research, in 2017, *we reviewed existing implementation research frameworks focusing on those applied in LMICs to study implementation strategies, identification of contextual factors, and implementation outcomes 8, 16– 19 . *We focused on identifying frameworks that had been used in LMICs to date which were relevant to health systems interventions, through a rapid review in PubMed with search terms “implementation science” and “LMICs.” We also reviewed publications in Implementation Science from the previous three years and conducted outreach and discussion with experts in the field at the time.* We identified relevant frameworks to develop a hybrid framework, which was adapted during the case study process to reflect emerging areas where changes were needed."

# Peer review: Reviewer response for version 1

**DOI:** 10.21956/gatesopenres.14432.r31232

**Published:** 2021-10-12

**Authors:** Ejemai Amaize Eboreime

**Affiliations:** 1 Department of Psychiatry, Faculty of Medicine and Dentistry, University of Alberta, Edmonton, AB, Canada

This is a really interesting contribution to the body of knowledge on the application of implementation science methods, theories, models and frameworks in global health.

 The authors have very well highlighted a gap which is increasingly being pointed out by many researchers and actors in global health:

**Most implementation science frameworks were designed for western contexts. Adaptations for use in global health are necessary.**

 I do have a few comments which will make this article stronger and more replicable.

 Methods:

***Mixed-methods vs multi-methods***

 The authors state that they used "mixed methods case study to leverage existing quantitative data on selected implementation outcomes (reach, fidelity, adoption) and equity with the qualitative sources describing strategies, contextual factors and selected outcomes (acceptability, feasibility). The Standards for Reporting Implementation Studies (StaRI) were used where relevant and is included in the Extended data"

1. I know this varies across schools of thought but I wonder whether this was not rather a multi-methods study. I see that at least two systematic reviews were conducted, qualitative interviews were also conducted, and the authors make reference to analysing quantitative data
2. It is not clear how quantitative methods were used in this study. The methods and results sections do not reflect this adequately
3. Given the multi-methods approach of this study, I believe that the StaRI is not sufficient as a checklist for this study. Could the authors also consider the COREQ and PRISMA checklists as well to ensure that the components of the qualitative and systematic review processes are covered?

***Development of the amenable U5M implementation research framework***

 The authors state that they *"reviewed existing implementation research frameworks focusing on those applied in LMICs to study implementation strategies, identification of contextual factors, and implementation outcomes"*

 However, there is no information on how this was carried out. Was there a systematic search strategy? This is important because several publications have emerged recently in an attempt to bridge this same gap. Were these emerging frameworks part of the review or was this a selective review process. This is not clear.

 Documentation of this process is very important for replication. Particularly as the authors posit in the results section that " *We found that no single framework covered the scope of the work from identification of EBIs to target leading causes of U5M, to selection and testing of strategies to achieve sustainability at scale, understanding the full range of potential contextual factors and exploring outcomes beyond the traditional coverage and effectiveness measures used in evaluation of health systems."*

 Such inference can only be made if all available frameworks were reviewed systematically. Thus, it would be very helpful for the authors to detail this search process.

 The only search strategy detailed seems to be around the EBI.

***Key informant interviews***

 It would be helpful to provide further details on who was interviewed. Also, it is not very clear in the results section what the outcomes of these interviews were.

Is the work clearly and accurately presented and does it cite the current literature?

Partly

If applicable, is the statistical analysis and its interpretation appropriate?

Not applicable

Are all the source data underlying the results available to ensure full reproducibility?

Partly

Is the study design appropriate and is the work technically sound?

Partly

Are the conclusions drawn adequately supported by the results?

Partly

Are sufficient details of methods and analysis provided to allow replication by others?

No

Reviewer Expertise:

Implementation Science, Global health

I confirm that I have read this submission and believe that I have an appropriate level of expertise to confirm that it is of an acceptable scientific standard, however I have significant reservations, as outlined above.

# Response

**Published:** 2021-11-16

**Authors:** Amelia VanderZanden

**Affiliations:** University of Global Health Equity, USA

We are grateful for your close review and comments on this article and have responded to each comment below as well as in the manuscript.

***Methods***

*The authors state that they used "mixed methods case study to leverage existing quantitative data on selected implementation outcomes (reach, fidelity, adoption) and equity with the qualitative sources describing strategies, contextual factors and selected outcomes (acceptability, feasibility). The Standards for Reporting Implementation Studies (StaRI) were used where relevant and is included in the Extended data"*

*I know this varies across schools of thought but I wonder whether this was not rather a multi-methods study. I see that at least two systematic reviews were conducted, qualitative interviews were also conducted, and the authors make reference to analysing quantitative data.*

Response: Thank you for your comments and identifying areas which were less clear.

 We understand that this could be a multi-methods, but because we used the qualitative methods to explain the quantitative findings (explanatory mixed methods), we have opted to keep the mixed methods terminology.

*It is not clear how quantitative methods were used in this study. The methods and results sections do not reflect this adequately*

Response: We used quantitative methods to look at the implementation outcomes of reach (coverage) and where possible, although limited, fidelity and equity. Because this coverage was our main metric of success, we used this to drive our qualitative work to help explain the quantitative results, implementation strategies, and contextual factors. We have added the following sentence to the Extraction and Analysis section of the Methods: “Reach, also defined as coverage, was calculated from the Demographic and Health Surveys (DHS).” We have also expanded on the quantitative results we report under implementation outcomes, excerpted below:

 “For example, PCV vaccine coverage was 94.7% in 2014 with a wealth gap of 5%. Measles vaccine coverage rose from 86.9% in 2000 to 95.2% in 2014, with the wealth equity gap hovering around 5% (4% in 2000, 6% in 2014). HIV testing and counseling during ANC was above 90% in 2014 with a wealth equity gap for both measures at 3%. Facility-based delivery had a higher wealth gap, but it decreased substantially from 43% in 2000 to 13% in 2014, with coverage increasing from 26.5% to 90.7% during that period.”

*Given the multi-methods approach of this study, I believe that the StaRI is not sufficient as a checklist for this study. Could the authors also consider the COREQ and PRISMA checklists as well to ensure that the components of the qualitative and systematic review processes are covered?*

Response: Thank you for the suggestions. We did not use PRISMA as this was a rapid review and not a systematic review. We agree that the COREQ checklist was a reasonable one to include and have added it here, in the extended data.

*The authors state that they "reviewed existing implementation research frameworks focusing on those applied in LMICs to study implementation strategies, identification of contextual factors, and implementation outcomes."* *However, there is no information on how this was carried out. Was there a systematic search strategy? This is important because several publications have emerged recently in an attempt to bridge this same gap. Were these emerging frameworks part of the review or was this a selective review process. This is not clear.*

*Documentation of this process is very important for replication. Particularly as the authors posit in the results section that *" *We found that no single framework covered the scope of the work from identification of EBIs to target leading causes of U5M, to selection and testing of strategies to achieve sustainability at scale, understanding the full range of potential contextual factors and exploring outcomes beyond the traditional coverage and effectiveness measures used in evaluation of health systems."* *Such inference can only be made if all available frameworks were reviewed systematically. Thus, it would be very helpful for the authors to detail this search process. The only search strategy detailed seems to be around the EBI.*

Response: Thank you for your question. The work on framework development was done in 2017. The authors were focused on implementation research frameworks which had been used in LMICs at that time. We conducted a search for implementation science in LMICs as a rapid review. We reviewed implementation science journals, as well as searching for IS frameworks in LMICs, and through outreach and discussion with experts in the field at the time.

 In terms of the newly emerging frameworks, we are excited by this growing interest. At the time that this framework was initially developed there were much fewer publications. We do refer to emerging work for example the Means et al article focused on expanding CFIR for LMICs, in the discussion, and have added the below (new text underlined here for emphasis): “For example, a recent review of uses of the CFIR framework found modifications needed to be more applicable for use in these settings with similar results from work using RE-AIM , highlighting emerging use of IR and where additional work is needed.”

***Methods/Results***

*It would be helpful to provide further details on who was interviewed.*

Response: Thank you for this comment. We have put together a table of type/role of KI for Rwanda to provide further detail. This is now Table 2 in the paper and is included here at bottom for reference.

*Also, it is not very clear in the results section what the outcomes of these interviews were.*

Response Thank you for your comment. We have worked to make this more clear. We extracted information from the interviews to explain the coverage and understand the implementation strategies and qualitatively other implementation outcomes. The interviews were also important in identifying contextual factors which served as facilitators (such as culture of data use) or barriers, and specific strategies (such as donor coordination and accountability). The data from these interviews was also combined with results from the literature and reports on EBI implementation to capture strategies, contextual factors, and implementation outcomes. We have added the below expanded detail on the outcomes of interviews in analysis to the results (in the section titled, Applying the Framework to Understand U5M Reduction in Rwanda): **“**Guided by the framework, we extracted evidence of the implementation outcomes for EBIs from the available literature and quantitative data, including appropriateness, acceptability, feasibility, effectiveness, coverage or reach, and equity, where possible. We also used the framework to develop an initial set of codes for EBIs, implementation outcomes, and contextual factors. Coding was done manually using directed content analysis with additional codes added based on identification of new concepts, factors, or strategies. These results were analyzed using an explanatory mixed methods starting with the EBI coverage data by the team and reviewed through a stakeholder meeting in Rwanda to provide feedback and validation, and identify transferable lessons with the potential for adoption or adaptation in other countries with challenges in reducing U5M.”

**Table 2. Rwandan key informants interviewed**

**Organization/Level**

**Number of key informants interviewed**

 Global/national including donors

 5 (33%)

 Government including Ministry of Health and Rwanda Biomedical Center

 6 (40%)

 Project managers and implementers

 3 (20%)

 Other partners

 1 (7%)

# Peer review: Reviewer response for version 1

**DOI:** 10.21956/gatesopenres.14432.r30732

**Published:** 2021-07-06

**Authors:** Betregiorgis Zegeye

**Affiliations:** 1 HaSET Maternal and Child Health Research Program, Shewarobit Field Office, Shewarobit, Ethiopia

The article entitled “Development and application of a hybrid implementation research framework to understand success in reducing under-5 mortality in Rwanda” [version1; peer review: awaiting peer review] is methodologically innovative and appreciable works. It is a well prepared draft. Few minor comments are given below.

**General comment**

 I think authors used very long sentences even up to six or more lines throughout the text, may lead readers to not easily understand the meanings. Therefore, I recommend shortening the sentences without changing the concept.

**Abstract**

 First line, please check the grammar as describe or should it read as described.

**Background**

 Last paragraph: line 1-4, do you believe the sentence after “amenable U5M” or “Deaths preventable through health system delivered evidence-based intervention (EBIs) defined amenable U5M? What about under-five death preventable through other than health system EBIs?

 Last paragraph: line 1 ^st^ and 4 ^th^ as mentioned in above comment; please check the grammar as describe or should it read as described.

**Methods**

**Desk review**: line 3 from the bottom, authors captured many contextual factors during desk review, but mentioned some of those factors were beyond the study’s scope. Can you mention those factors that are beyond the study’s scope?

**Desk review:** What was the inclusion and exclusion criteria during desk review?

**Desk review:** line 12-17 have repeated paragraph, please remove one without citation.

**Key informant interview** (line 7), in the sentence “…..MOH employees responsible for overall U5M efforts, or specific disease or intervention areas” is a little confusing. It seems they had been working to facilitate U5M. So, please revise it.

                                                       

**Key informant interview** (Second paragraph, line 11), please specifically mention which type of consent authors were obtained; verbal or written?

**Key informant interview** (Second paragraph, line 12), authors mentioned about recordings, but not clearly stated weather audio or video recordings are used.

**Key informant interview** (Second paragraph, line 19), revise the typo error (principle in to principal).

 On Figure 2, to keep the logical flow the phrase “curative and preventive” better to be changed in to “preventive and curative”.

**Results**

**Cross cutting contextual factors**; line 5 needs revision.

 Authors used women empowerment and female empowerment interchangeably (e.g., figure 2, desk review line 9 from the bottom, discussion 3 ^rd^ paragraph: line 4), but they are not same and need to be select one and use throughout the texts.

**Discussion**

 Second paragraph (line 2), the first sentence ended with”…across many if not all of the EBIs”, need revision.

Is the work clearly and accurately presented and does it cite the current literature?

Yes

If applicable, is the statistical analysis and its interpretation appropriate?

Yes

Are all the source data underlying the results available to ensure full reproducibility?

Yes

Is the study design appropriate and is the work technically sound?

Yes

Are the conclusions drawn adequately supported by the results?

Yes

Are sufficient details of methods and analysis provided to allow replication by others?

Yes

Reviewer Expertise:

NA

I confirm that I have read this submission and believe that I have an appropriate level of expertise to confirm that it is of an acceptable scientific standard.

# Response

**Published:** 2021-11-16

**Authors:** Amelia VanderZanden

**Affiliations:** University of Global Health Equity, USA

We are grateful for your close review and comments on this article and have responded to each comment below as well as in the manuscript.

***General comment***

*I think authors used very long sentences even up to six or more lines throughout the text, may lead readers to not easily understand the meanings. Therefore, I recommend shortening the sentences without changing the concept.*

Response: Thank you for your feedback, we agree the long sentences are challenging and have worked to shorten throughout the article.

***Abstract***

*First line, please check the grammar as describe or should it read as described.*

Response: Thank you for your comment. We believe it should remain “describe” as we are explaining what we will do in the article

***Background***

*Last paragraph: line 1-4, do you believe the sentence after “amenable U5M” or “Deaths preventable through health system delivered evidence-based intervention (EBIs) defined amenable U5M?*

Response: The sentence describes the focus of our study, which was specifically deaths preventable through health system-delivered EBIs.

*What about under-five death preventable through other than health system EBIs?*

Response: U5 death preventable through other than health system EBIs are important as well but were not the focus of the work described in this paper.

***Background***

*Last paragraph: line 1st and 4th as mentioned in above comment; please check the grammar as describe or should it read as described.*

Response: Thank you for your comment. Again, we believe it should remain “describe” as it pertains to what is covered in the article and is not discussing the research work done (which is in the past tense).

***Methods***

*Desk review: line 3 from the bottom, authors captured many contextual factors during desk review, but mentioned some of those factors were beyond the study’s scope. Can you mention those factors that are beyond the study’s scope?*

Response: Thank you for your question. The authors stated that in-depth reviews of the *implementation* of these interventions not directly led by the health sector were beyond the scope of the research – specifically of interventions including health system strengthening, education, female empowerment, reproductive health, poverty reduction, improving water and sanitation, and programs designed to improve nutritional status beyond management of acute severe malnutrition. We treated them as contextual factors which may have facilitated or hindered implementation of the targeted EBIs (health system delivered targeted U5M directly) but not as a focus of the implementation research into how they were implemented.

*Desk review: What was the inclusion and exclusion criteria during desk review?*

Response: We conducted a rapid review with no consort diagram. Inclusion criteria included anything that included one of the EBIs from the list (Extended Data Tables 1a and 1b), included qualitative or quantitative information on contextual factors, implementation strategy, or implementation outcomes, and was specific to the country of focus. We have edited the text in the desk review section to make this more explicit.

*Desk review: line 12-17 have repeated paragraph, please remove one without citation.*

Response: Thank you for pointing this out as it appears to have been added during the typesetting. We have made this correction.

*Key informant interview (line 7), in the sentence “.....MOH employees responsible for overall U5M efforts, or specific disease or intervention areas” is a little confusing. It seems they had been working to facilitate U5M. So, please revise it.*

Response: We have revised to include the work “reduction”, as below (underlined here for ease of viewing): “current and former Ministry of Health employees responsible for overall U5M reduction efforts, or focusing on specific diseases or intervention areas.”

*Key informant interview (Second paragraph, line 11), please specifically mention which type of consent authors were obtained; verbal or written?*

Response: Thank you for your comment. A statement about consent was included in the ethical approval and consent to participate section. We have revised the text to clarify that it was verbal consent.

*Key informant interview (Second paragraph, line 12), authors mentioned about recordings, but not clearly stated weather audio or video recordings are used.*

Response: We have revised to specify that they were audio recordings.

*Key informant interview (Second paragraph, line 19), revise the typo error (principle in to principal).*

Response: This typo has been corrected.

*On Figure 2, to keep the logical flow the phrase “curative and preventive” better to be changed in to “preventive and curative”.*

Response: We agree with this edit and have made the change in the figure.

***Results***

*Cross cutting contextual factors; line 5 needs revision.*

Response: We have reviewed the text and made the correction to include the missing close parenthesis on line 3.

*Authors used women empowerment and female empowerment interchangeably (e.g., figure 2, desk review line 9 from the bottom, discussion 3rd paragraph: line 4), but they are not same and need to be select one and use throughout the texts.*

Response: Thank you for flagging this, you are correct that these are not interchangeable. We have revised to “female empowerment” in Figure 2 for consistency.

***Discussion***

*Second paragraph (line 2), the first sentence ended with”...across many if not all of the EBIs”, need revision.*

Response: Thank you for your comment. We have changed the sentence from “Applying this framework also supported the extraction of implementation strategies that were common across many if not all of the EBIs.” to now read (underlined here for ease of reading the changed text): “Applying this framework also supported the extraction of implementation strategies that were used in the work to introduce and expand access across most or all of the EBIs.”
